# Exploring Patterns of Disturbed Eating in Psychosis: A Scoping Review

**DOI:** 10.3390/nu12123883

**Published:** 2020-12-18

**Authors:** Nicolette Stogios, Emily Smith, Roshanak Asgariroozbehani, Laurie Hamel, Alexander Gdanski, Peter Selby, Sanjeev Sockalingam, Ariel Graff-Guerrero, Valerie H. Taylor, Sri Mahavir Agarwal, Margaret K. Hahn

**Affiliations:** 1 Centre for Addiction and Mental Health (CAMH), Toronto, ON M6J 1H3, Canada; nicolette.stogios@mail.utoronto.ca (N.S.); emilycc.smith@mail.utoronto.ca (E.S.); roshanak.asgariroozbehani@mail.utoronto.ca (R.A.); laurie.hamel@camh.ca (L.H.); peter.selby@camh.ca (P.S.); sanjeev.sockalingam@camh.ca (S.S.); ariel.graff@camh.ca (A.G.-G.); mahavir.agarwal@camh.ca (S.M.A.); 2 Institute of Medical Science (IMS), University of Toronto, Toronto, ON M5S 1A8, Canada; 3 Department of Human Biology, University of Toronto, Toronto, ON M5S 3J6, Canada; alexander.gdanski@mail.utoronto.ca; 4 Department of Family and Community Medicine, University of Toronto, Toronto, ON M5G 1V7, Canada; 5 Dalla Lana School of Public Health, University of Toronto, Toronto, ON M5T 3M7, Canada; 6 Department of Psychiatry, University of Toronto, Toronto, ON M5T 1R8, Canada; 7 Bariatric Surgery Program, University Health Network, Toronto, ON M5T 2S8, Canada; 8 Department of Psychiatry, University of Calgary, Calgary, AB T2N 1N4, Canada; valerie.taylor3@albertahealthservices.ca

**Keywords:** food intake, eating behaviour, diet, overconsumption, binge eating, weight gain, obesity, hedonic pathway, homeostatic pathway

## Abstract

Disturbed eating behaviours have been widely reported in psychotic disorders since the early 19th century. There is also evidence that antipsychotic (AP) treatment may induce binge eating or other related compulsive eating behaviours. It is therefore possible that abnormal eating patterns may contribute to the significant weight gain and other metabolic disturbances observed in patients with psychosis. In this scoping review, we aimed to explore the underlying psychopathological and neurobiological mechanisms of disrupted eating behaviours in psychosis spectrum disorders and the role of APs in this relationship. A systematic search identified 35 studies that met our eligibility criteria and were included in our qualitative synthesis. Synthesizing evidence from self-report questionnaires and food surveys, we found that patients with psychosis exhibit increased appetite and craving for fatty food, as well as increased caloric intake and snacking, which may be associated with increased disinhibition. Limited evidence from neuroimaging studies suggested that AP-naïve first episode patients exhibit similar neural processing of food to healthy controls, while chronic AP exposure may lead to decreased activity in satiety areas and increased activity in areas associated with reward anticipation. Overall, this review supports the notion that AP use can lead to disturbed eating patterns in patients, which may contribute to AP-induced weight gain. However, intrinsic illness-related effects on eating behaviors remain less well elucidated, and many confounding factors as well as variability in study designs limits interpretation of existing literature in this field and precludes firm conclusions from being made.

## 1. Introduction

Psychosis is the hallmark feature of various psychiatric illnesses, including schizophrenia (SCZ), schizoaffective disorder, schizophreniform disorder and bipolar disorder [1]. It is a severely debilitating condition with an estimated worldwide prevalence of approximately 4.6 per 1000 people [2]. The American Psychiatric Association and World Health Organization have conceptualized psychosis as consisting of altered perception and impaired reality testing, including positive symptoms such as hallucinations and delusions [3]. Severe mental illnesses additionally are associated with cognitive deficits and negative symptoms, which can drive functional impairment and illness associated disability [4,5].

Antipsychotic (AP) medications are currently the cornerstone treatment for psychotic disorders [6]. Unfortunately, APs are associated with serious metabolic adverse effects [7], which increase patients’ risk of developing metabolic syndrome, type 2 diabetes, and cardiovascular disease (CVD). Notably, CVD is the leading cause of premature mortality in severe mental illness, reducing life expectancy by 11–20 years [8,9,10]. While clozapine and olanzapine carry the greatest metabolic liability [11], all AP medications cause weight gain in younger patients with limited previous AP exposure [12]. Similarly, these medications have been shown, independently of class or individual agent, to increase risk of type 2 diabetes in patients with SCZ [10].

Weight gain, a common consequence of AP treatment, occurs when there is a positive energy balance, meaning that energy intake exceeds energy expenditure [13]. Beyond the metabolic effect of APs, weight gain in psychotic disorders is also, in part, explained by unfavorable behaviours. For instance, patients with SCZ may have higher intake of calorie dense foods and lower intake of healthy foods than the general population [14]. Other contributing factors include lower levels of physical activity and significantly higher rates of smoking and alcohol consumption [15]. All these behaviours are also associated with lower socioeconomic status and higher unemployment among patients with SCZ [15,16]. Furthermore, epidemiological reviews have suggested that approximately 10% of patients with SCZ suffer from binge eating disorder (BED) or night eating syndromes, which is five times higher than in the general population [17]. Thus, disturbed eating behaviour may also contribute to the significant weight gain and metabolic disturbances experienced by these patients.

Looking beyond social, environmental and behavioural factors, energy homeostasis is controlled by intricate physiological pathways. Patients with SCZ may have subclinical metabolic dysregulations including dyslipidemia [18], hyperglycemia and insulin resistance [19] present at the earliest stages of the illness, which are further exacerbated by AP therapy [20,21]. Furthermore, impaired regulation of appetite related hormones including elevated insulin (linked with insulin resistance) and low leptin and adiponectin (secreted by adipose tissue) levels are also implicated in the pathophysiology of weight gain in psychosis spectrum disorders [18,22]. Ghrelin, which stimulates hunger, does not appear to be altered in AP-naïve or largely unmedicated first episode psychosis (FEP) patients [18]; however, olanzapine use may be associated with decreased ghrelin levels, which is a similar phenomenon to what is observed in obesity [23].

**Figure 1:**
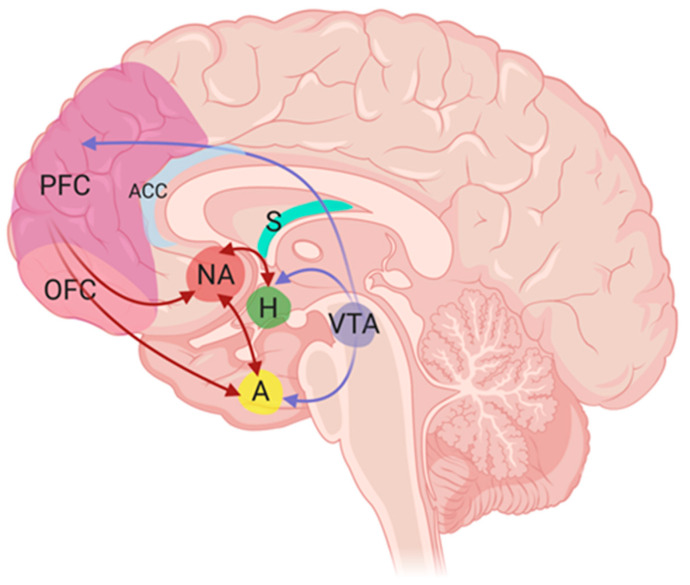
Limbic and reward pathways involved in eating behaviour and appetite. Eating behavior is closely associated with activity of the reward circuitry of the brain, which involves a group of neuronal structures that become activated and release dopamine when exposed to rewarding stimuli like food [27]. The pathway most associated with reward circuitry of the brain is referred to as the mesolimbic dopamine pathway, which starts with production and release of dopamine in the ventral tegmental area (VTA). The mesolimbic dopamine pathway then relays VTA signaling to the nucleus accumbens (NA), an area associated with motivation. The other aspect of the reward system is known as the mesocortical pathway which connects the VTA to the prefrontal cortex (PFC). This region also includes the orbitofrontal cortex (OFC), a key area involved in cognitive processes, such as decision making and memory. The PFC additionally forms connections with sensory and limbic pathways as well. Importantly, the reward pathway is activated, both before and after receipt of a reward suggesting that dopamine increases reward seeking behavior. Thus, any disruption of these pathways could potentially lead to disordered eating behavior. The VTA is also functionally and anatomically connected to the hypothalamus (H), primarily the lateral hypothalamus. The hypothalamus integrates homeostatic signals from various peripheral organs along with reward responses to modulate food intake and energy expenditure according to changes in metabolic state [28]. The arcuate nucleus of the hypothalamus (not shown), where neuropeptide Y (orexigenic) and proopiomelanocortin (anorexigenic) producing neurons reside, is the main area responsible for energy sensing and eating behavior. VTA, ventral tegmental area; NA, nucleus accumbens; H, hypothalamus; PFC, prefrontal cortex; OFC, orbitofrontal cortex; A, amygdala; ACC, anterior cingulate gyrus; S, striatum.

While the physiological homeostatic mechanisms underlying altered eating patterns in this population have been the subject of recent meta-analyses and reviews [18,20], less is known about the psychopathological and neurobiological mechanisms that may be implicated in the non-homeostatic regulation of food intake. Non-homeostatic eating behaviour involves the hedonic and reward aspects of food intake that is separate from the physiological drive stimulated by energy requirement [24]. This aspect of eating behaviour is regulated by the reward system, which includes the mesolimbic dopamine circuit (involving the ventral tegmental area and nucleus accumbens), as well as nuclei in the amygdala and hippocampus that are interconnected to the hypothalamus and brainstem (the latter implicated in homeostatic feeding regulation) [25]. Disruption at any level of these complex neural networks regulating eating behaviour may be implicated in the weight gain and metabolic sequalae associated with SCZ. Moreover, these disruptions are likely to involve aspects intrinsic to SCZ, and/or associated with AP treatment [26]. The reward and limbic pathways involved in eating behavior and appetite are depicted in Figure 1 in more detail.

Given the high metabolic comorbidity observed in psychosis spectrum disorders, elucidating the psychopathological and neurobiological mechanisms underlying disrupted eating behaviours is crucial in helping to improve both the physical and psychological well-being of patients. In this scoping review, we aim to provide a comprehensive overview of disordered eating behaviours observed in psychosis spectrum disorders. We synthesize evidence from clinical studies employing self-report questionnaires and surveys to measure changes in food intake, craving and appetite, as well as behavioural neuroimaging studies to further explore the neurobiological mechanisms underlying these disturbances in eating patterns. In an attempt to distinguish illness intrinsic effects from those caused by treatment with APs, we present separately, when possible, results from studies examining AP-naïve patients (vs. matched healthy controls), and healthy controls (HCs) or AP-naïve patients beginning APs.

## 2. Methods

Our protocol was developed using the scoping review methodological framework proposed by the Joanna Briggs Institute [29]. The objectives, inclusion criteria and methods for this scoping review were specified in advance and documented in a protocol.

### 2.1. Search Strategy

An a priori search strategy was developed and tested in consultation with the Education and Liaison Librarian for the Institute of Medical Science at the University of Toronto. Databases searched included Ovid MEDLINE, Ovid EMBASE, Ovid PsychINFO, EBSCO’s CINAHL, CENTRAL on Wiley and Scopus. A grey literature search was also performed by mining references from relevant articles and review papers identified in the search, as well as searching SCOPUS for conference proceedings. Vocabulary and syntax were adjusted across databases. There were no language, date or methodology restrictions, with the exception of case studies and opinion pieces, which were excluded from the results. The specific search string for each database can be found in Supplementary Table S1.

### 2.2. Source of Evidence Screening and Study Selection

Article screening, including automatic duplicate removal, was completed using Covidence [30]. Two authors independently screened and assessed titles and abstracts (NS and AG), while another two independently completed the full-text screening (ES and RA). Conflicts were resolved by discussion and consensus between the authors and in consultation with the senior authors (SMA and MH). At all stages, screening decisions were made according to prespecified inclusion and exclusion criteria which are outlined in Table 1.

### 2.3. Charting the Data

A data extraction template was created and piloted among study authors (NS, ES, RA) and was refined and finalized based on data extracted from a sample of studies. The information displayed in Table 2 was extracted from each included full-text article.

### 2.4. Synthesis and Presentation of Results

Studies were summarized and presented according to their relevant category: (1) Studies describing eating patterns, food preferences and diet composition using dietary recall, food diaries and food frequency questionnaires; (2) studies measuring self-reported appetite, hunger and/or satiety using a mix of validated questionnaires and semi-structured interviews (see Table 3); and (3) studies using neuroimaging methodologies to assess neurobiological changes in relation to aspects of eating or food intake. A narrative summary of each study is reported in its respective subsection, with overlap in other subsections if applicable. Where appropriate, tables were created to concisely summarize characteristics of included studies and relevant findings (see Tables 4–6).

## 3. Results

### 3.1. Search Results

**Figure 2:**
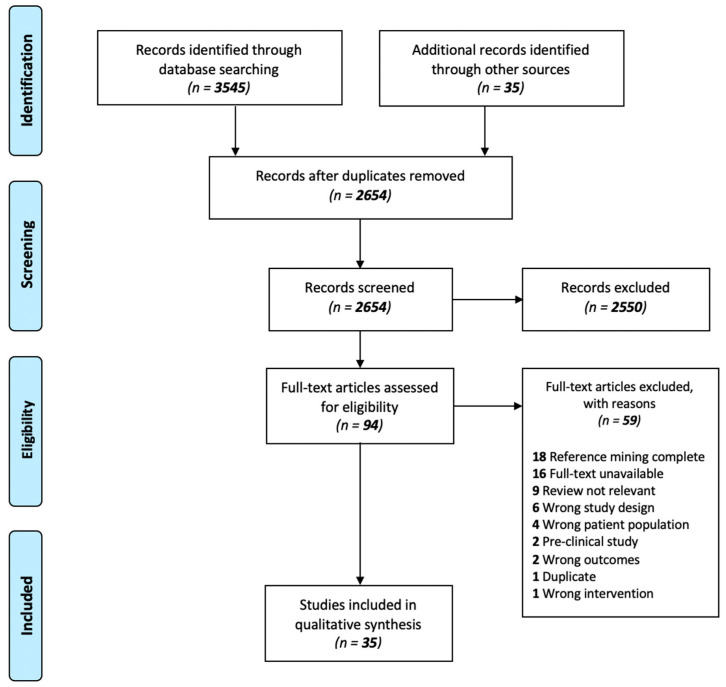
Preferred reporting items for systematic reviews and meta-analyses (PRISMA) flowchart. Literature search and selection process of included studies.

Our initial search revealed 3545 results, which was reduced to 2654 after removal of duplicates. Following title and abstract screening, 94 studies were assessed for full-text eligibility. A total of 35 studies that considered dietary composition, food preference and cravings and/or eating patterns in patients with SCZ or HCs exposed to APs were deemed eligible and included in our qualitative synthesis (Figure 2; preferred reporting items for systematic reviews and meta-analyses (PRISMA) flow diagram).

The studies identified in our search used a number of validated methodologies and questionnaires to examine different aspects of eating behavior. The most commonly employed subjective dietary assessments include food diaries, 24-h dietary recall, the Three Factor Eating Questionnaire (TFEQ), the Dutch Eating Behavior Questionnaire (DEBQ), visual analog scales (VAS), the Food Craving Inventory (FCI), the Food Craving Questionnaire (FCQ) and the Food Frequency Questionnaire (FFQ). The TFEQ addresses three aspects of eating behaviour including restriction of food intake, loss of control of food intake and responsivity to internal hunger cues. Previous studies in the general population indicate that increased body weight is positively associated with TFEQ scores [31,32,33], particularly disinhibition and susceptibility to hunger [34,35,36]. The DEBQ is a self-report questionnaire designed to assess different factors regulating eating behaviour including desire to restrict food intake, tendency to eat in response to emotions and responsivity to external cues. Overweight and obese individuals generally display greater scores in all DEBQ domains compared to normal weight individuals [37,38,39], with the most robust relationship found for the emotional eating factor [37,39,40]. General hunger and appetite rating scales (VAS, and Likert scales) are also frequently employed to assess eating behaviour [41], while the FCQ and FCI are used to measure general and specific food cravings, respectively. A more detailed description of these questionnaires can be found in Table 3.

Our search yielded 9 studies that described dietary composition (summarized in Section 1 below); 19 studies that looked at eating patterns and food-related cognitions (summarized in Section 2 below); and 7 studies that used neuroimaging methodologies (summarized in Section 3 below). In order to facilitate elucidation of the specific effects of illness vs. APs, we have divided the results within each of the three methodology-based sections into three subsections based on population type: Patients only, patients (specifying AP-naïve cohorts) vs. controls and HCs exposed to APs.

### 3.2. Findings from Subjective Food Preference and Dietary Composition Studies

We retrieved nine studies that measured dietary composition and food preference using 24-h dietary recall, food diaries and the Food Frequency Questionnaire (FFQ) [53]. Only two studies indicated that part of the patient population studied were AP-naïve, although no subgroup analyses for these patients were available [54]. Table 4 summarizes the characteristics of included studies in this section, along with main findings.

#### 3.2.1. Patients vs. Healthy Controls

Seven of the included dietary composition studies compared patients with healthy controls [55,56,57,58,59,60,61]; of these studies, only three matched patients to HCs according to key baseline features, such as age, sex and BMI [55,56,58].

Three cross-sectional studies [57,58,59] revealed that patients consumed significantly more total calories per day than HCs. However, results regarding specific dietary composition (carbohydrates, fat, protein) were less consistent, with the authors reporting either increased protein consumption and decreased saturated fat consumption by patients [58], decreased protein consumption and a trend towards increased saturated fat [57] or no difference between patients and controls [59]. Gattere et al. (2018) noted a trend towards increased scores on the FCQ with increasing psychopathology (psychotic disorders > at risk mental states > controls), suggestive of a relationship between food cravings and disease state, while Nunes et al. (2014) found no significant association between body mass index (BMI) and antipsychotic type (FGA, SGA).

The three remaining case-control studies also noted differences in nutritional patterns between patients and HCs, including increased fat consumption and more frequent snacking in patients [57,62]. Interestingly, these studies also stratified their results by sex, revealing differences in dietary composition and eating behaviour such as snack preference and calorie intake. Details of the differences between males and females are reported in Table 4. Beyond sex effects, Stefanska et al. (2017) also found that in the patient group, lower caloric intake was associated with lower BMI, waist circumference, waist-to-hip ratio and body fat content [60].

The final study included in this section explored eating behaviour differences between HCs and patients with SCZ on OLA treatment. This study revealed that that 70% of the OLA-treated patients reported ingesting a significantly greater amount of food than usual, with no compensatory increase in physical activity levels [56].

#### 3.2.2. Patients Only

Only one dietary composition study explored the effects of APs on food intake and energy expenditure in patients [54]. The study was conducted in males only and compared patients treated with olanzapine to those treated with haloperidol. After four weeks, the olanzapine group experienced a significant increase in BMI and caloric intake, but no difference in dietary composition, energy expenditure or physical activity level. Important to note is that, similar to the aforementioned study by Eder et al. (2001) [56], physical activity levels were low [54], suggesting that olanzapine may lead to weight gain through a combination of increased caloric intake and decreased physical activity.

#### 3.2.3. Healthy Controls Only

Consistent with the patient-only studies discussed above, an HC study conducted by Fountaine et al. revealed that volunteers randomized to receive olanzapine gained more weight and consumed significantly more calories than those randomized to placebo [53]. Interestingly, this weight gain was accompanied by an increase in resting energy expenditure and a trend towards increased physical activity in the olanzapine group, which the authors hypothesize may have occurred to compensate for the increase in caloric intake.

### 3.3. Findings from Subjective/Self-Report Questionnaires on Appetite, Satiety and Craving

In total, there were 19 studies [50,62,63,64,65,66,67,68,69,70,71,72,73,74,75,76,77,78,79] that examined differences in eating behaviour, subjective appetite and food craving using self-reported questionnaires and interviews. Table 5 presents a detailed summary of these studies. Seven studies specifically considered DSM-IV diagnostic and research criteria for eating disorders (EDs) including binge eating disorder (BED); Section 2.1) [62,63,64,65,66,67,68], while the remainder of the studies assessed subjective appetite and/or eating-related cognitions (Section 2.2) [50,69,70,71,72,73,74,76,77].

#### 3.3.1. Binge Eating and Other Eating Disorder-Related Behaviours

Seven studies [62,63,64,65,66,67,68] explored the occurrence of binge-eating symptomatology in patients being treated with SGAs. In all cases, binge eating symptomatology was determined based on DSM-IV research criteria for BED unless otherwise specified.

#### 3.3.2. Patients Only

Consistent with the dietary composition studies discussed above, all five patient studies [63,64,65,66,67] found that treatment with clozapine or olanzapine increased appetite, food intake, food craving and/or risk of weight gain in non-FEP patients. Interestingly, the studies further suggest that these changes may be related to AP-mediated induction of binge eating. For example, one study [63] found that 17% of patients reported episodes of binge eating after starting clozapine, with one patient seeing remittance and re-occurrence of binge eating after discontinuing and then restarting treatment. In another study, the authors found that half of all included clozapine- and olanzapine-treated patients screened positively for binge eating behaviour (BE group), with over half reporting *onset* of episodes of binge eating during the current medication regime [66]. A similar retrospective clozapine/olanzapine study [64], found that 14% of patients met DSM-IV diagnostic criteria for an ED, specifically eating disorders not otherwise specified (including BED) or bulimia nervosa. Subsequent comparison of scores from the Questionnaire on Eating and Weight Patterns QEWP [51] and adverse drug reaction (ADR) scale [81] revealed that ED onset was “definitely” or “probably” linked to AP exposure. Prospective studies also appear to support a relationship between clozapine/olanzapine treatment and binge eating, with one showing a significant increase in binge eating episodes from baseline to endpoint [65], and another identifying a positive correlation between olanzapine-induced appetite increases and behaviours similar to DSM-IV BED criteria such as “preoccupation with food” and ”eating until uncomfortably full” [67].

#### 3.3.3. Patients vs. Controls

Similar to the findings mentioned above, two case-control studies conducted by Khazaal et al. found evidence of a link between psychosis and disordered eating. In their first study [62], the authors observed altered self-esteem and self-control, greater fear of weight gain, and a greater desire to control weight in patients with SCZ compared to controls as determined by a revised version of the Mizes Anorectic Cognitive Questionnaire (MAC-R). They also found that females had higher MAC-R scores than men, suggestive of sex and/or gender effects. In the second study [68], they found a significantly higher prevalence of DSM-IV binge eating symptoms and BED in overweight/obese patients with SCZ compared to weight-matched controls.

### 3.4. Subjective Appetite, Hunger and Satiety

Our search identified 12 studies [50,69,70,71,72,73,74,76,77] that used self-report measures including visual analog scales (VAS), the TFEQ and the DEBQ to measure subjective appetite/hunger and eating-related cognitions.

#### 3.4.1. Patients Only

Conclusions from longitudinal studies regarding the effects of AP medications (particularly SGAs) on appetite were mixed. For example, two studies, one in which patients were randomized to receive olanzapine or risperidone [71], and another where patients were randomized to either disintegrating or standard olanzapine tablets [70] found no significant effect of AP treatment on appetite (Eating Behaviour Assessment and VAS) with a non-significant trend towards decreased appetite. In contrast, two different studies found significant weight-related changes in eating behaviour following AP exposure. In particular, Ryu et al. found that SGA treatment increased weight as well as subjective hunger, appetite and food craving (Drug-Related Eating Behaviour Questionnaire; DR-EBQ) [50]. On the other hand, despite failing to report overall longitudinal changes, Garriga et al. (2019) observed interesting moderating effects of baseline BMI, stage of illness and sex in clozapine-treated patients [69] (see Table 5). The authors also found a significant positive correlation between specific food cravings (FCI) and subsequent consumption (Cuestionario de Frecuencia de Consumo de Alimentos; CFCA), suggesting that psychological desire may translate into behavioural changes.

In the only cross-sectional study identified, Sentissi et al. compared eating behaviour between AP-naïve or AP-free, FGA-treated and SGA-treated patients with SCZ [72]. They found that BMI status was positively associated with TFEQ disinhibition (significant) and hunger (nearing significance) scores. Furthermore, SGA-treated patients showed greater reactivity to external eating cues (DEBQ) than the FGA-treated, but not the untreated patients.

#### 3.4.2. Patients vs. Healthy Controls

All five studies comparing patients and controls were cross-sectional studies. Generally, there were mixed results regarding group differences in appetite/satiety, which highlights a need for longitudinal studies in this area.

In one study, although patients experienced increased hunger (VAS) and decreased satiation compared to HCs following a standardized meal [74], the groups did not differ in spontaneous intake and food preference during a buffet-type meal three hours later. The authors also found that patients had increased TFEQ scores in all three domains (cognitive restraint, disinhibition, susceptibility to hunger), a finding that remained significant after controlling for BMI. A separate study exploring executive functioning (which is known to be impaired in SCZ), found that patients displayed significantly worse delay of gratification and executive functioning than HCs in a task involving food reinforcement [76]. These impairments were associated with increased restrained eating behaviour and disinhibition, as well as increased BMI, suggesting that disease-related dysfunction in the dorsolateral prefrontal cortex (DLPFC) and dorsal anterior cingulate cortex (ACC) (prefrontal-ACC network) may increase susceptibility to overeating, thereby promoting weight gain.

In contrast to the studies discussed above, Schanze et al. (2008) found no group differences between patients with SCZ, patients with major depressive disorder, and HCs in any of the TFEQ domains [77]. Furthermore, they observed no effect of medication class (AP, antidepressant, no medication) on TFEQ scores [77]. Similarly, Abbas et al. (2013) found no significant difference in food craving (FCI) between AP-treated patients with SCZ and HCs [73]. Finally, Folley et al. (2010) found that patients and HCs did not differ in their response time or food preference when asked to choose between two food images [75]. Interestingly, although patients generally gave higher positive ratings to food stimuli than HCs, instances when they gave lower ratings were correlated with increased anhedonia. This led the authors to suggest that while preference judgements appear to be intact in patients, the hedonic value they place on food may be altered.

#### 3.4.3. Controls Only

Our search retrieved two randomized, double-blind, placebo-controlled studies in HCs examining subjective appetite/hunger following short-term SGA exposure. In the first study, Roerig et al. (2005) found that two weeks of either olanzapine or risperidone exposure led to weight gain compared to placebo, although only olanzapine reached statistical significance [78]. The authors also observed a trend towards both greater food intake (kcal/day) and an increase in appetite (measured using a 100 mm VAS) in the olanzapine group relative to the other groups. In contrast, Teff et al. (2015) observed no significant change in weight, subjective hunger/fullness or calorie intake following nine days of SGA exposure [79]. Importantly, in contrast to the aforementioned HC study by Fountaine (2010), neither study reported significant changes in physical activity or energy expenditure in association with AP treatment.

### 3.5. Findings from Neuroimaging and Brain Structure Studies

Our search yielded seven studies that used neuroimaging methodologies to study food preference and eating behavior in patients with SCZ. The characteristics of these studies and a summary of their main findings can be found in Table 6. Six studies used functional magnetic resonance imaging (fMRI) along with visual analog scales (VAS) and/or eating questionnaires [82,83,84,85,86,87] and one study used structural MRI to study brain morphology [88]. One study was conducted on AP-naïve (*n* = 22) patients [88], and one was conducted on patients who were AP-naïve (*n* = 9) or had been medication free for at least six weeks (*n* = 20) [86] (Section 3.2).

#### 3.5.1. Patients Only

A study by Stip (2015) and colleagues compared brain activity (fMRI) in response to videos of food in patients with SCZ before and after initiating or switching to olanzapine therapy [84]. The authors found that 16 weeks of olanzapine exposure led to significantly decreased neuronal activation in the salience network (SN), an important network involved in reward processing and reward anticipation. Specific regions affected by olanzapine included the anterior fronto-insular (aFI) cortex, amygdala, thalamus and anterior cingulate cortex (ACC). The decrease in SN activation was associated with a decrease in dietary restraint (TFEQ), leading the authors to suggest that AP-mediated disruptions of the SN may promote changes in eating behaviour.

#### 3.5.2. Patients vs. Healthy Controls

In an earlier publication (conducted in the same cohort as the 2015 study [84], but including a HC comparator), Stip et al. (2012) used static food images and examined subjective appetite (VAS) and TFEQ scores in patients with SCZ before and after starting olanzapine [85]. Using fMRI, they found that 16 weeks of olanzapine treatment led to a significant increase in activation in the supplementary motor area, right fusiform gyrus, insular cortex, amygdala and parahippocampal regions in response to static food images. Comparing these changes in activation to controls, it was found that neural activity in the premotor area, somatosensory cortices and bilaterally in the fusiform gyri of patients with SCZ was normalized, while activity in the insular cortices, amygdala and cerebellum was ‘overshot’. Interestingly, this hyperactivation was positively correlated with disinhibition (TFEQ), suggestive of an association between OLA-induced increases in brain activity and dysfunctional processing of food-related stimuli.

An earlier study, using the same patient cohort (but pre-switch to olanzapine) as the two aforementioned studies by Stip and colleagues [84,85], similarly assessed brain activity (fMRI) in response to food cues [83]. Relative to HCs, patients with SCZ showed increased activation in brain regions involved in action planning and regulation of homeostatic signals including the red thalamic nucleus, left parahippocampal gyrus and left middle frontal gyrus. Furthermore, the authors found that activity in the red thalamic nucleus was positively correlated with cognitive restraint (TFEQ Factor 1), while activity in the left middle frontal gyrus was associated with increased disinhibition (TFEQ Factor 2). This led them to suggest that cortical processes may disrupt or override sub-cortical hypothalamic appetite regulation signals in patients with SCZ. Additional correlational analyses controlling for either AP dose (chlorpromazine CPZ equivalents) or disease severity (Positive and Negative Syndrome Scale; PANSS) revealed a significant positive correlation between AP dose and susceptibility to hunger (TFEQ Factor 3) and a significant negative correlation between PANSS score and cognitive restraint. This led to the conclusion that both SCZ and AP medications may contribute to appetite dysregulation in patients, but through different mechanisms.

In a similar but independent fMRI study, Grimm et al. (2012) asked chronic patients with SCZ and HCs to rate their appetite levels on a VAS following presentation of neutral or appetitive stimuli [82]. Even after adjusting for body weight and AP dose (CPZ equivalents), patients were found to have significantly weaker activation in the dorsal striatal region (post appetitive stimulus vs. neutral images) compared to controls. In keeping with the findings by Stip et al. 2012 [84], these results led the authors to suggest that SCZ may involve intrinsic disruptions in the SN, leading to altered reward anticipation and eating behavior. However, despite these functional differences (and in contrast to some of the studies already discussed), Grimm et al. found no significant difference in appetite between patients and controls.

#### 3.5.3. First Episode Patients vs. Controls

In a structural MRI study, Emsley and colleagues [88] investigated morphological brain changes after 13 weeks of AP treatment (risperidone or flupentixol injections) in AP-naïve FEP patients with SCZ, in relation to changes in BMI and metabolic indices. Regions of interest included the ventral diencephalon (vDC) and prefrontal cortex (PFC), which respectively represent key homeostatic and hedonic food intake regulatory areas. As there were no differences in MRI or metabolic outcomes between AP treatment groups, patients from both groups were pooled together for analysis. The authors found that compared to HCs, patients experienced a volume reduction in the vDC (a region containing the hypothalamus), which was strongly correlated with BMI and glucose increases and dyslipidemia. In contrast, no changes were observed in the PFC region, leading the authors to suggest that acute AP treatment primarily results in disruption of homeostatic functions (and not reward pathways). However, following post-hoc testing, these volume reductions were no longer significant and increased volumes in the control group were reported, which the authors attributed to random fluctuations due to small sample size.

In a recent fMRI study, Borgan et al. (2019) investigated neural responsiveness to appetitive stimuli in untreated FEP patients and HCs [86]. Comparing fMRI blood oxygen level dependent (BOLD) signaling response to appetitive stimuli between groups, the authors found that patients consistently exhibited the same regional patterns of neural activity observed in controls, indicative of normal neural responses to food cues. This led them to suggest that neural processing of food may be unaltered in the early stages of the illness and may instead be influenced by AP treatment.

### 3.6. Healthy Controls Only

We retrieved one neuroimaging study in HCs, which examined the effects of seven days of olanzapine administration on fMRI responses to visual stimuli (appetitive and neutral) as well as to receipt of an actual food reward [87]. Olanzapine treatment resulted in increased appetite as measured by both liquid breakfast intake and TFEQ scores (particularly disinhibition). This was accompanied by increased activation in brain regions involved in the reward pathway in response to both anticipation (inferior frontal cortex, striatum and ACC) and receipt (caudate, putamen) of appetitive stimuli. Interestingly, they also observed a concurrent decrease in activation in the lateral orbitofrontal cortex, which is thought to be involved in satiety.

## 4. Discussion

We performed a scoping review, which aimed to explore associations between psychosis spectrum disorders, food consumption, and disruptions in appetite and eating behaviors. Our search retrieved 35 studies, which we subsequently organized into three sections based on main theme or methodology: (1) Food composition and dietary preference, (2) patterns of eating behaviour and subjective appetite and (3) neural correlates of appetite and eating behavior. These sections are discussed individually, followed by a discussion of postulated mechanisms, and a more general discussion of limitations and future directions of this field.

### 4.1. Food Composition and Dietary Preference

The studies identified in our search provide evidence that overconsumption, in the form of both increased frequency and quantity of food consumption, differs between patients and HCs [57,58,59,60], which may contribute to the high rates of obesity in patient populations. In keeping with the general population, lower calorie intake among patients is associated with lower BMI, waist circumference, waist-to-hip ratio and body fat content [60]. Furthermore, dietary preference appears to be sex-specific [55,60,61], which could explain the differential propensity for weight gain among male and female SCZ spectrum disorder patients [89].

Disentangling the extent to which observed differences in caloric intake and dietary composition relate to biological factors intrinsic to the illness and/or AP treatment is challenging. While work in AP-naïve FEP populations can be helpful in delineating intrinsic illness related factors, only two of the dietary composition studies we retrieved included AP-naïve individuals [56,57]. However, subgroup analyses comparing HCs and AP-naïve patients were not performed, precluding inferences on dietary alterations that may primarily result from intrinsic illness effects. Unfortunately, it is similarly difficult to delineate the relative effects of APs on diet as studies in HCs indicate either no significant difference [78,79] or a significant increase in caloric intake [53] following SGA exposure.

It is also important to consider socioeconomic, environmental, and lifestyle factors that may precipitate a snowball effect on unhealthy dietary patterns among patients. Patients with psychosis spectrum disorders tend to belong to lower socioeconomic status (SES) groups [15,90]. This in turn relates to their ability to afford or have access to a nutritious diet. Notably, none of the dietary composition studies we reviewed matched patients to HCs in terms of SES, including income and education level. Three cross-sectional studies did, however, report significant differences in socio-demographic variables of patients vs. controls [58,60,61]. As such, it is possible that psychosocial stress related to socioeconomic factors, or symptoms of psychosis, may influence food intake in patients. Chronic stress has also been associated with hyperphagia [91] and preference for palatable foods [92]. Thus, failure to match patients to HCs according to key demographic features such as SES is a potential source of variation and should be considered in future studies.

### 4.2. Eating Behaviour, Cravings and Subjective Appetite

Synthesis of the studies identified in our search revealed a positive association between BMI/weight and altered appetite, hunger and/or food cravings in patients with psychosis spectrum disorders [50,64,65,69,72], as well as between SGA treatment and binge eating symptomatology [62,63,64,65,66,68]. Similar to what is observed in the ED literature [93,94], two studies also noted a relationship between restrictive eating behaviour (high restraint and high disinhibition scores) and increased consumption and weight gain among patients [72,74]. This may potentially suggest a common mechanism between EDs and the disordered eating patterns seen in psychosis patients.

In addition, APs may increase appetite and response to both internal and external hunger cues (as assessed by the TFEQ), putting patients at higher risk of overeating and subsequent weight gain [54,56,74,83,89]; however, the literature appears quite contradictory [70,71,73,82]. Potential explanations for these discrepancies could be choice of rating scale or questionnaire [95] and experimental conditions (i.e., fasting state, meal challenge and type), which differed widely across studies. As such, it is difficult to determine the relative contribution of illness vs. AP drugs on appetite.

Longitudinal HC studies also provide mixed evidence regarding the effects of APs on appetite and eating behaviors. Some studies indicate increased appetite, body weight and food intake following olanzapine treatment, indicative of a potential causal link [53,87], while other studies indicate SGA exposure does not significantly affect appetite or food intake despite inducing weight gain [78] and metabolic changes such as insulin resistance [79]. The latter point may suggest that central insulin and/or leptin resistance resulting from AP-induced weight gain and increases in adiposity may lead to appetite change, rather than appetite driving weight change [79].

### 4.3. Neural Correlates of Appetite and Eating Behavior

A variety of neuroimaging strategies have been employed to examine neurobiological mechanisms implicated in food intake patterns in patients, with a majority of the work (six out of seven retrieved studies) focusing on functional changes captured by fMRI in response to appetitive cues. Unfortunately, though, the different behavioural paradigms and brain regions of interest of each study made it difficult to draw any broad conclusions or generalizations. Two studies suggest that APs may contribute to disrupted appetite regulation and eating behaviour by increasing activation in areas involved in action planning and homeostatic signals [85], and regions implicated in cognitive and motivational processing of food [83]. However, these findings appear limited to static appetitive stimuli as dynamic stimuli led to decreased activation of the SN [84]. Interestingly, changes in regional activation correlated with disinhibition (TFEQ) scores across all three studies [83,84,85]. Similarly, AP treatment in HCs appears to [87] enhance activation in the brain reward circuitry, and decrease activation in the lateral orbital frontal cortex, consistent with loss of inhibitory effects on eating behaviour.

In determining the relative effects of illness vs. AP treatment, one AP-naïve study did not report any neural differences between patients or controls, indicating that food-related neural processing is not intrinsically dysregulated in SCZ [86]. In contrast, a different study found that chronic patients with SCZ on stable AP therapy exhibited significantly reduced activation in striatal regions involved in reward processing, an association that persisted even after controlling for AP dose. This suggests that the neural alterations involved in appetite regulation may be related to factors intrinsic to SCZ, which become more prominent as the illness progresses, and further exacerbated by AP therapy. This is consistent with structural MRI findings, which found that AP treatment reduced the volume of the vDC, but not the PFC in AP-naïve FEP patients [88].

### 4.4. Postulated Neurobiological Mechanisms Involved in Appetite/Feeding Regulation

**Figure 3:**
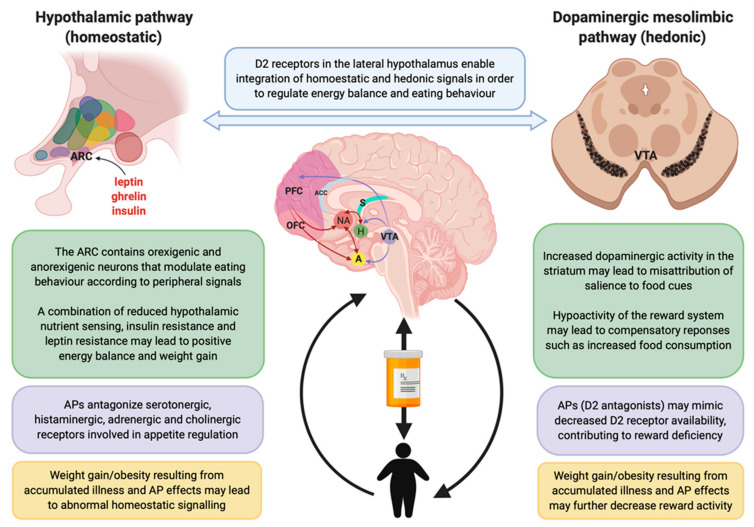
Overview of the homeostatic and hedonic pathways regulating appetite and eating behaviour. Text in green boxes describe the function of each pathway and implications for regulation of eating behaviour; text in purple boxes describes antipsychotic-induced effects; text in yellow boxes describes how weight gain affects pathway function. Abbreviations: VTA, ventral tegmental area; NA, nucleus accumbens; H, hypothalamus; PFC, prefrontal cortex; OFC, orbitofrontal cortex; A, amygdala; ACC, anterior cingulate gyrus; S, striatum; AP, antipsychotic; D2 = dopamine 2.

While the contributing effects of intrinsic illness related factors vs. those of AP medications remain difficult to separate, existing theoretical frameworks may provide a neurobiological rationale for the differences in eating behaviours and appetite between patients with pychosis spectrum disorders and HCs. The postulated disruptions in hedonic/motivational and homeostatic mechanisms in patients with pyschosis spectrum disorders are summarized in Figure 3.

### 4.5. Hedonic Reward Mechanisms

The mesolimbic dopamine reward system is instantiated by a network of brain structures innervated by dopaminergic projections from the ventral tegmental area (VTA), including the nucleus accumbens (NAc), hypothalamus, amygdala, and PFC regions [96,97] (see Figure 1). Mesolimbic dopamine has primarily been implicated in the incentive motivational dimension of reward, including reward prediction [98], and the attribution of motivational salience to reward-related cues (associated with the concept of ‘wanting’ or ‘craving’) [99].

In turn, increased dopaminergic transmission in the striatum is a core neurobiological feature of SCZ that responds to first line AP treatment [100,101]. The striatum integrates inputs received from the majority of the cortex and projects to the mesolimbic dopamine system and cortical salience networks [102]. Its role has been associated with making inferences about the current state of the environment [103], whereas abnormal dopaminergic reactivity in the striatum may lead to misattribution of salience to external or internal cues relating to food or appetite.

Moreover, reward hypoactivity, which is related to negative symptoms of SCZ [104,105], may result in compensatory responses such as increased food consumption to achieve sufficient rewarding stimulation [82,106]. Furthermore, as function in the dorsal striatum is believed to be modulated by body weight, metabolic dysregulations accumulated throughout the course of the illness and perturbated by AP therapy may also be implicated in reduced striatal activity, similar to what is seen in obese individuals [106].

Additionally, disrupted function in the DLPFC, ACC and mediodorsal nucleus of the thalamus has been associated with impaired executive function in SCZ [107]. Analogous to observations of diminished executive function in the obese population, this may lead to poorer choices in food selection or difficulty in inhibiting responses to cravings. Consistent with this, several studies included in this review suggest that patients with SCZ have increased disinhibition [72,74,83,84,85], and an increased incidence of binge eating [59,67,68,72,81,108], which may reflect deficits in executive function related to prefrontal-ACC dysfunction [107].

The limited body of literature reporting on AP-naïve FEP patients precludes direct attribution of any dysregulations to inherent illness factors. However, APs share the uniting property of dopamine 2 (D2) receptor antagonism, which may mimic decreased D2 receptor availability, and thus contribute to the reward deficiency/overcompensation phenotype. Indeed, a relationship between reduced D2 receptor function and reward dysfunction has already been observed in obesity [109]. Beyond their effects on the dopamine system, APs also interact with serotonergic, histaminergic, adrenergic, muscarinic and cholinergic receptors, all of which are differentially involved in appetite modulation [84,110]. As such, the role of APs in disturbed eating behaviours is likely complex, involving widespread regions of the brain and signaling networks, with additional interfaces with illness-related disruptions in these pathways.

### 4.6. Homeostatic Mechanisms

Homeostatic mechanisms of food regulation are thought to be primarily regulated by the hypothalamus, a region anatomically situated to confer accessibility to hormones (leptin, ghrelin, insulin) and nutrients (glucose, fatty acids) in the blood and cerebral spinal fluid (CSF) to relay information about the body’s energy stores to the brain [28] (see Figure 3). The topic of impaired hormonal regulation of feeding in SCZ has been the subject of a recent comprehensive review, supporting that early disruptions in these pathways likely progress over the course of illness and are further exacerbated by APs [20]. These homeostatic pathways are also thought to interact with dopamine reward circuits to regulate eating behavior and energy balance [111], potentially mediated by the high concentration of D2 receptors in the lateral hypothalamus [20] Thus, it is possible that the connections between these pathways may be altered in SCZ. For example, Stip et al. (2012) found evidence of increased signaling in the amygdala, a key limbic structure responsible for integrating homeostatic signals with extrinsic influences to modulate eating behavior [85]. However, this field of research is not well developed and is likely further complicated by the interaction between intrinsic aspects of psychosis spectrum disorders, AP treatment and psychological or environmental factors [112,113]. Interestingly, one study included in our review demonstrated volume reductions in the hypothalamus of AP-naïve patients following olanzapine treatment [88]. However, the relevance of changes in hypothalamic size in relation to obesity and metabolic disorders as well as the effects of AP treatment in relation to brain volume changes are controversial [114,115]. Further research combining advanced neuroimaging approaches (functional and structural) with food cues and stimuli relevant to hedonic and non-hedonic aspects of eating and assessments of hormonal activity is needed.

### 4.7. Strengths, Limitations and Future Directions

A key strength of this scoping review is that the search was broad, allowing for a comprehensive overview of the current state of the literature pertaining to eating behaviours and food consumption in psychosis spectrum disorders. Moreover, to the best of our knowledge, this is the first review to summarize the findings of neuroimaging studies that sought to elucidate the neurobiological mechanisms underlying eating behaviours among psychosis spectrum patients.

Nevertheless, there are some limitations which must be addressed. First, our search revealed high heterogeneity in both study design and questionnaires employed, which made comparing studies difficult and precluded conclusions from being made. Second, the majority of studies used subjective self-report measures of appetite/craving, results of which may be influenced by factors outside of hunger [58]. Additionally, the use of patient recall, as in the case of food diaries or during retrospective interviews, may lead to inaccurate estimations of food intake [57]. This is particularly relevant given that recall is known to be impaired in SCZ [58]. As alluded to by others, future studies that use both subjective and objective measures of appetite (e.g., calorie intake) [71], complemented by neuroimaging approaches [82] are required to move the field forward. Furthermore, only one fMRI study examined the effect of somatosensory (gustatory) stimuli on appetite and eating preferences [87]; the remaining five studies focused solely on visual processing of food-related cues, potentially missing key mediators of altered eating behaviour [116].

Importantly, very few of the studies identified in our search considered AP-naïve FEP patients, with the vast majority involving patients who had previously been exposed to AP therapy. This makes it difficult to determine whether any abnormal eating patterns observed in patients are intrinsic to the illness or secondary to the effects of APs. Additionally, while HC studies are a good way to remove the confounding effect of illness, they preclude identification of any interaction between intrinsic dysfunction in eating and AP effects. Prospective studies in which AP-naïve patients are exposed to APs would be particularly useful in exploring this illness-treatment interaction. Moreover, it should also be considered that studies in chronic patients with SCZ are confounded by cumulative illness associated lifestyle factors and treatments, which may affect both eating patterns and weight gain [7,117]. Further to this point, once obesity and other metabolic comorbidity is established, this may have secondary effects on physiology of feeding regulation [28]. Finally, metabolic consequences of AP treatment are known to be most pronounced in AP-naïve or FEP patients, suggesting that this may represent the critical period to capture early changes in eating behavior and appetite, which drive early weight gain [118]. Unfortunately, at present, the temporal course or trajectory of disordered eating in psychosis cannot be determined as most studies did not report trends over multiple timepoints. This would be a point worth considering when designing future longitudinal studies.

Finally, many of the studies comparing patients with HCs did not match groups on key sociodemographic and physiological (i.e., BMI, gender/sex) factors (see Table 4, Table 5 and Table 6), constituting a significant confound. To this last point, while sex emerged as an important mediator of appetite and feeding disruptions in some of the studies included in this review, the majority of studies did not account for sex. This is highly relevant given that in the general population, global obesity rates differ for males and females (10% and 18%, respectively [119]), as do TFEQ and DEBQ scores [36,120,121]. Furthermore, in SCZ, females seem to be more at risk for AP-induced metabolic disturbances than males [122,123]. Further investigation is therefore warranted to determine whether sex-related differences in eating behaviors can explain this increased vulnerability.

## 5. Conclusions

While disruptions in hormones involved in homeostatic mechanisms of appetite control in patients with pychosis spectrum disorders have been the subject of several reviews and meta-analyses, our scoping review highlights the behavioral and neurobiological underpinnings of altered eating behaviour in this population. Our synthesis of evidence from food surveys and self-report questionnaires generally supports the notion that patients with pychosis spectrum disorders exhibit increased appetite and craving for fatty food, increased caloric intake and increased frequency of (over) consumption, which may be associated with increased disinhibition. Early evidence also suggests that disturbed eating behaviours in this population could be mediated by abnormal processing of food-related stimuli within neural systems related to the mesolimbic reward circuit. In addition, it is possible that impaired cognitive restraint and executive functioning intrinsic to psychosis may make patients more susceptible to developing disordered eating patterns in response to weight gain and/or increased appetite and cravings. Future prospective studies with larger samples and AP-naïve populations are needed to improve the evidence base in this field and help dissect the intrinsic and extrinsic illness factors involved in disturbed appetite regulation. This will have important implications for development of pharmacological and behavioral interventions which, by targeting cardiometabolic comorbidities, may have the potential to increase patient life span and improve overall quality of life.

## Figures and Tables

**Table 1:** List of inclusion and exclusion criteria for selected studies.

Inclusion Criteria	Exclusion Criteria
Schizophrenia or other Schizophrenia Spectrum Disorders First episode patients (FEP) Chronic AP-naïve Psychosis, psychotic disorders Bipolar disorder Eating disorders Bulimia nervosa Binge eating disorder Night eating syndrome Food addiction Behavioral studies Neuroimaging Questionnaires Antipsychotics First Generation Antipsychotics (FGA)/typical Second Generation Antipsychotics (SGA)/atypical	Any psychiatric diagnosis not listed in the inclusion criteria (e.g., major depression disorder, anxiety disorders) included as the primary population of interest Off-label AP use Opinion pieces, letters Treatment studies of APs for Anorexia Nervosa At-risk/subclinical psychosis/psychotic-like experiences included as primary population of interest Purely physiological studies (i.e., no behavioural/appetite measures) Pre-clinical studies (mice/rodent, other non-human animals)

**Table 2:** List of information extracted from each full-text article meeting inclusion criteria.

Extracted Data
Author(s) Year of publication Origin/country or ethnicity of participants Aims/purpose Type of study Population and sample size within the source of evidence (if applicable) Population demographics (Sex/gender, age) Methodology/methods Intervention type, comparator and details of these (e.g., duration of the intervention) (if applicable). Duration of the intervention (if applicable) Outcomes and details of these (e.g., how measured) (if applicable) Key findings that relate to the scoping review question/s and concepts

**Table 3:** Description of self-report questionnaires used to measure subjective appetite and eating behaviour; ‘Original Source’ indicates the authors who originally developed the questionnaire.

Name	Original Source	Description	Subscales and Other Relevant Information
Three-Factor Eating Questionnaire (TFEQ)	Stunkard and Messick, 1985 [42]	51-item questionnaire measuring three aspects of eating behaviour (cognitive restraint, disinhibition, hunger)	Cognitive dietary restraint: active attempt to restrict caloric intake and control body weight Disinhibition: likelihood of overeating when exposed to a favorable environment (e.g., palatable food) or stress Susceptibility to hunger: susceptibility of individuals to perceive hunger and ingest food as a result; can be triggered by internal and external stimuli High disinhibition and hunger scores associated with increased weight, obesity [34,35] and overconsumption/binge-eating (DSM-IV/DSM 5 criteria) [43,44])
Dutch Eating Behaviour Questionnaire (DEBQ)	Van Strien et al., 1986 [40]	33-item questionnaire measuring three factors that regulate eating behaviour (restraint, emotion, external factors)	Restrained eating: the desire or intention to restrict food intake; correlated with TFEQ cognitive restraint Emotional eating: the tendency to eat in response to negative emotions; associated with overconsumption and obesity; positively correlated with TFEQ disinhibition [35,38,45] External eating: the tendency to eat in response to environmental triggers and food cues; differs between overweight and normal weight individuals but does not appear to be predictive of weight gain [37,39,45]
Visual Analog Scale (VAS)	Stubbs et al., 2000 (review) [46]	Psychometric tool used to quantify subjective appetite	The most common method used in appetite research includes a horizontal (100-mm) line anchored by two extremes such as “not at all hungry/as hungry as I’ve ever felt” Sensitive to experimental manipulations (e.g., dietary composition, levels of food intake, medication effects) Best used in conjunction with more objective measures such as calorie intake or physiological changes
Food Craving Questionnaire (FCQ)	Cepeda-Benito et al., 2000 [47]	Questionnaire measuring general food cravings (trait and state version)	FCQ-Trait (FCQ-T): 9 factors measuring general food cravings and tendencies FCQ-State (FCQ-S): 5 factors measuring food cravings in response to situational factors Higher cravings are associated with binge eating and overconsumption FCQ scores are positively correlated with TFEQ disinhibition and hunger scores
Food Craving Inventory (FCI)	White et al., 2002 [48]	Questionnaire measuring specific food cravings (carbohydrates, sweets, fats, fast-food fats)	Considers both intensity and frequency of food cravings, where craving is defined as “an intense desire to consume a particular food (or food type) that is difficult to resist” Fat craving associated with increased BMI Positively correlated with TFEQ disinhibition and hunger scores (stronger effect for hunger)
Drug-Related Eating Behavior Questionnaire (DR-EBQ)	Lim et al., 2008 [49]	14-item questionnaire that quantifies changes in appetite, craving and eating behaviour after beginning antipsychotic treatment	Statements cover feelings of hunger, pre-occupations with food, desire to eat, control of eating and food cravings [50] For each statement, patients indicate whether the frequency of behaviour/cravings have increased since starting AP therapy using a Likert scale
Questionnaire on Eating and Weight Patterns (QEWP)	Spitzer et al., 1993 [51]	Questionnaire used to evaluate the presence of binge-eating symptomatology and binge-eating related disorders	Developed using DSM-IV criteria for BED/BN and validated using clinical assessments Can be used to diagnose BED and BN (purging or non-purging type) Statements cover social impairment, body image, history of dieting, comorbidities (e.g., depression, alcohol/drug abuse, sexual abuse), compensatory behaviours
Mizes Anorectic Cognitions Questionnaire—Revised (MAC-R)	Mizes et al., 2000 [52]	24-item questionnaire used to assess eating disorder-related cognitions (anorexia nervosa, bulimia nervosa, binge eating disorder)	Covers three aspects: weight regulation, approval and self-control; statements rated on a 5-point Likert scale (higher scores represent greater dysfunction)

**Table 4:** Characteristics of studies reporting on dietary composition.

Study	Study Description					Main Significant Results
	Design/Aim	Sample (Size, Diagnosis), Mean Age (Years), Mean BMI (kg/m^2^)	Sex (% F), Race/Ethnicity (%)	Illness Duration/Previous AP Exposure	Assessments	
Amani 2007 [55]	Cross-sectional (case-control) Dietary preference in patients with SCZ compared to HC	30 SCZ inpatients, 16–76 years Age: 32.3 (M), 32.5 (F) BMI: 22 (M), 26 (F) 30 HCs (matched for age, sex) Age: 35.6 (M), 36.6 (F) BMI: 25.6 (M), 25.4 (F)	SCZ: 37% F HC: 47% F	Illness duration: at least one year; previous AP exposure not stated	Dietary recall (FFQ) Food Guide Pyramid to calculate dietary scores	Females: • Dietary scores ↓ and percent body fat ↑ for patients than HCs. • Consumed ↑ carbonated drinks, but ↓ milk, vegetables and nuts daily than the HCs • Males: • Patients ate ↑ hydrogenated fats and full-fat cream, but ↓ red meats, vegetable oils and nuts servings per day than HCs • Male patients ate ↑ vegetables, eggs, cream and chocolate than female patients
Eder 2001 [56]	Longitudinal (8 weeks) Association of olanzapine induced weight gain with an increase in body fat	10 SCZ in patients treated with OLA monotherapy Dose range: 7.5–20 mg/d Age: 30.4 BMI: 22.4 10 HCs (matched for age, sex) Age: 35.2 BMI: 22.1	SCZ: 20% F HC: 20% F	No APs prior to OLA: 5 Previous APs: 5 (flupentixol, fluphenazine, risperidone, or haloperidol)	Semi-standardized structured interview to assess changes in eating behaviour and physical activity	70% of patients reported they ingested a significantly greater amount of food than usual during a period of time throughout the study No change in physical activity
Fountaine 2010 [53]	Randomized, placebo controlled, two treatment crossover study (15 + 15 days, 12-day washout between arms) Comparing food intake and energy expenditure following olanzapine vs. placebo in healthy men	30 male HCs (21 completers) Age: 27 (range: 18–49) BMI: 22.6	All Males	N/A	Food intake monitored and weighed REE, daily activity level	Mean total food intake in OLA group ↑ 18% (from 3860 kcal to 4230 kcal) relative to PBO Mean weight change with OLA: 4.1 kg 43.9% of patients experienced clinically significant weight gain (≥ 7%) Early significant weight gain after 2 months of therapy occurred in 23.4% of the patients ↑ REE and respiratory quotient with OLA compared to PBO
Gattere 2018 [57]	Cross-sectional Dietary intake in early psychosis	124 early psychotic disorder (PD), 82 (66.1%) FEP patients with <5 years from illness onset Schizophreniform: *n* = 22 Schizoaffective: *n* = 12 Psychotic disorder NOS: *n* = 70 Age: 24.7 BMI: 24.3 36 at-risk mental state (ARMS) Age: 22.2 BMI: 22.2 62 HCs (not matched) Age: 23.5 BMI: 22.2	PD: 34.7% F; 76.6% Caucasian, 9.7% Latino American, 8.1% Arabian, 4.0% Gypsy, 0.8% Black, 0.8% Asian ARMS: 27.8% F; 88.9% Caucasian, 8.3% Latino American, 2.8 Arabian HC: 48.4% F; 95.2% Caucasian, 3.2% Latino America, 1.6% Arabian	PD: Monotherapy: 72 (58.1%) RIS = 31 PAL = 13 OLA = 17 QUE = 1 ARI = 10 Combination: 33 (26.6%) No APs: 19 (15.3%) ARMS: Monotherapy: 7 (19.4%) RIS = 1 OLA = 3 ARI = 3 Combination: 3 (8.3%) No APs: 27 (75%)	24-h dietary recall Food Craving (FCQ-State) IPAQ-short form	Patients consumed ↑ calories/day and % of calories from saturated fatty acids than HCs Patients consumed ↓ protein than HCs Trend towards increased food craving scores (Food Craving Questionnaire; FCQ) with increasing psychopathology (psychotic disorders > at risk mental states > controls) Both patient groups (PD and ARMS) reported reduced physical activity compared to HCs
Gothelf 2002 [54]	Longitudinal (4 weeks) Food intake and weight gain in adolescent males with SCZ treated with OLA vs. HAL	20 male SCZ inpatients OLA: *n* = 10 (MD: 14 mg/d) HAL: *n* = 10 (MD: 6.5 mg/d) Age (both): 17 BMI (OLA only): 24.5	All Males	OLA: mean washout period = 17.6 days Drug naïve = 1 Clomipramine = 1 AP other than OLA = 8	Dietary Evaluation (2-day monitoring of food intake by dietician; food weighed) Daily energy expenditure, REE, physical activity	BMI ↑ greater for OLA than HAL ↑ caloric intake (27.7%) in OLA group. No changes in dietary composition (carbohydrates, fats, or proteins), REE or physical activity levels
Nunes 2014 [58]	Cross-sectional (case-control) Evaluating nutritional status, food intake and cardiovascular disease risk in SCZ patients	25 SCZ outpatients Age: 40.5 (range: 18–59) BMI: 29.09 25 HCs (matched for age, sex, BMI) Age: 37.2 BMI: 26.91 Total sample Age: 38.9 BMI: 28.0	SCZ: 40% F HC: 48% F	SGA = 68% FGA = 28% Both = 4%	Dietary recall (FFQ)	Patients consumed ↑ total calories, calories per kg body weight, protein per kg body weight, and % of carbohydrates and trans fatty acids Patients consumed ↓ saturated fat, unsaturated fat and omega−6
Strassnig 2003 [59]	Cross-sectional (case-control) Exploring potential causes of weight gain in SCZ patients compared to general population (NHANES III)	146 outpatients with psychosis SCZ, paranoid type: *n* = 69 Schizoaffective: *n* = 53 Psychotic disorder NOS: *n* = 24 Age: 43 BMI: 32.7 Patient data compared to general population (NHANES III)	47% F 54% White, 46% Black	NR	24-h dietary recall	Patients consumed ↑ total calories, fats and carbohydrates compared to general population (NHANES III). Relative proportion of each food group (carbohydrates, fat, protein) did not differ between groups.
Stefanska 2017 [60]	Cross-sectional Eating habits and nutritional status in patients with SCZ and affective disorders	60 SCZ, 18–67 years Age: 34.1 (M), 41.3 (F) BMI: 27.6 (M), 27.2 (F) 61 recurrent depressive disorder, 18–67 years Age: 38.0 (M), 46.4 (F) BMI: 26.1 (M), 26.7 (F) 98 HCs (not matched), Age: 33.0 (M), 43.0 (F) (range: 18–69 years) BMI: 27.3 (M), 25.8 (F)	SCZ: 53.3% F Depression: 54.1% F HCs: 61.2% F	AP treatment (FGA or SGA) for at least one year (AP type not specified) Age at onset: 23.3 (M), 30.1 (F) Illness duration (years): 9.5 (M), 10.4 (F)	24-h dietary recall Resting metabolic rate (RMR)	Patients consumed ↑ fat (with a predominance of saturated fatty acids over polyunsaturated fatty acids), and ↓ protein than HCs Lower energy intake promoted lower BMI, waist circumference, waist-to-hip ratio and body fat Female SCZ patients consumed ↑carbohydrates and % of energy from carbohydrates compared to female HCs Male SCZ patients consumed ↑ fat, particularly saturated fatty acids compared to male HCs
Stefanska 2018 [61]	Cross-sectional (case-control) Assessing the nutritional value males consumed by patients with SCZ	85 SCZ outpatients, 18–65 years Age: 37.8 (M), 39.0 (F) BMI: 25.0 (M), 25.1 (F) 70 HCs (not matched) Age: 35.9 (M), 38.2 (F) BMI: 25.9 (M), 24.4 (F)	SCZ: 52.9% F HC: 57.1% F	AP treatment (FGA or SGA) for at least one year (AP type not specified) 1 AP = 39% 2 or 3 APs = 61% Age at onset: 26.7 (M), 273 (F) Illness duration (years): 10.0 (M), 12.3 (F)	24-h dietary recall	↑ snacking in patients than HCs Female SCZ patients consumed ↑ calories and showed an ↑preference for sweets than HCs Male SCZ patients had ↓ energy intake and content of the majority of assessed nutrients compared to HCs

Note: All main findings reported in this table are statistically significant unless otherwise indicated. SCZ = Schizophrenia, BP = Bipolar Disorder. HC = Healthy controls, FEP: First episode psychosis, AMI = amisulpride, PBO = Placebo, OLA = olanzapine, PAL = Paliperidone, HAL = Haloperidol, RIS = Risperidone, QUE = Quetiapine, ZIP = Ziprasidone, CPZ = Chlorpromazine, FGA = First generation antipsychotics, SGA = Second generation antipsychotic, NR = Not reported, TFEQ = Three-Factor Eating Questionnaire, FCQ = Food Craving Questionnaire, FCI = Food Craving Inventory, VAS = visual analog scale, DR-EBQ = Drug-Related Eating Behaviour Questionnaire, FFQ = Food Frequency Questionnaire, EBA = Eating Behaviour Assessment, DEBQ = Dutch Eating Behaviour Questionnaire, QEWP = Questionnaire on Eating and Weight Patterns, MD = Mean Dose.

**Table 5:** Characteristics of studies reporting on subjective ratings of appetite, craving and hunger.

Study	Study Description					Main Significant Results
	Design/Aim	Sample (Size, Diagnosis), Mean Age (Years), Mean BMI (kg/m^2^)	Sex (% F), Race/Ethnicity	Mean Illness Duration/Previous AP Exposure (*n*)	Assessments	
Bromel 1998 [63]	Longitudinal (10 weeks) Effect of CLZ on food craving in patients with SCZ	12 SCZ in patients treated with CLZ (MD: 273 mg/d; range: 81–475 mg/d) SCZ: *n* = 9 FGA: *n* = 3 Age: 31 (range: 18–65) BMI: 25.8	50% F	Nine patients treated with psychotropic medication (including APs) prior to starting CLZ (type/duration of previous exposure not specified)	Binge eating/ED symptomatology (DSM-IV) Binary appetite/craving scale	CLZ treatment ↑ weight, BMI and adiposity 75% (9/12) of patients reported ↑ appetite/hunger and specific food cravings 17% (2/12) of patients reported onset of binge eating behaviour One patient saw remittance and re-occurrence of binge eating after discontinuing and then restarting CLZ
Gebhardt 2007 [64]	Longitudinal (retrospective) Binge-eating symptomatology associated with CLZ and OLA use in patients with psychosis	64 patients being treated for psychotic symptoms with CLZ or OLA SCZ: 52.3% SCZ spectrum disorder: 12.3% Mood disorder: 18.5% Substance abuse: 7.7% Personality disorder: 3.1% Other diagnoses: 7.7% Age: 30.7 (range: 13.3–64.6)	47% F	Patients were treated with CLZ or OLA for at least 4 weeks prior to inclusion in study CLZ: *n* = 33 OLA: *n* = 31	QEWP (DSM-IV binge-eating) Adverse drug reaction (ADR) scale Appetite (4-point Likert-type scale)	69% of patients experienced an increase in appetite after starting CLZ/OLA, with a stronger effect for CLZ Post-CLZ/OLA weight gain was associated with ↑ appetite 14% of patients met DSM-IV criteria for BED or bulimia nervosa ED onset was “definitely” or “probably” linked to CLZ/OLA exposure (Naranjo probability) Post-CLZ/OLA EDs were more common in patients with a history of EDs
Kluge 2007 [65]	Randomized, double blind, parallel (6 weeks) Effect of CLZ and OLA on food craving and binge eating in patients with SCZ spectrum disorders	30 SCZ (*n* = 26), Schizoaffective (*n* = 3), Schizophreniform (*n* = 1) inpatients, 18–65 years CLZ: *n* = 15 OLA: *n* = 15 Age: 36.7 (CLZ), 32.8 (OLA) BMI: 25.4 (CLZ), 24.4 (OLA) Dosing (last 4 weeks of study): mean modal dose = 266.7 mg (CLZ), 21.2 mg (OLA)	CLZ: 53% F OLA: 67% F	Age of illness onset: 30 (CLZ), 28 (OLA)	Binge eating/ED symptomatology (DSM-IV) Binary appetite/craving scale	CLZ/OLA treatment ↑ body weight and BMI 97% (29/30) of patients reported ↑ appetite and 90% (27/30) reported ↑ food intake Patients experienced ↑ binge eating and craving for sweet and fatty foods, with no significant effect of gender
Theisen 2003 [66]	Cross-sectional (BE vs. non-BE) with an exploratory retrospective analysis Comparing binge eating symptomatology in patients with SCZ treated with clozapine and olanzapine	74 SCZ inpatients CLZ: *n* = 57 OLA: *n* = 17 Age: 19.8 (range: 15.6–26.6) Two sub-groups based on prevalence of binge eating behaviour Binge eating (BE): *n* =37 Non-binge eating (non-BE): *n* = 37	36% F	Illness duration (years): 2.4 (range = 0.3–7.1)	QEWP (DSM-IV binge-eating)	26% (7/27) of females and 11% (5/47) of males met lifetime criteria for full BED or BN In the BE group, 54% (20/37) of patients reported onset of binge eating episodes during the current CLZ/OLA regime Patients in the BE group had ↑ current BMI and experienced ↑ weight gain from medication onset to time of study compared to those in the non-BE group
Treuer 2009 [67]	Longitudinal (6 months) Food intake and nutritional factors associated with weight gain in patients with SCZ and BD treated with OLA	622 SCZ or BD outpatients (589 completers) treated with OLA (MD: 11.4 mg/d; mean duration during study: 5.4 months) SCZ: 85% of sample BD: 15% of sample Age: 32.6 years BMI: 23.2	56% F Multinational (China, Romania, Mexico, Taiwan) 61% East Asian, 25% Caucasian, 14% Hispanic	Lifetime AP exposure: 74.5% of sample (duration not specified) Past 6 months: 45.2% of sample	Interview to assess appetite (5-point Likert), frequency of food consumption, subjective energy levels and physical activity	44% of patients experienced clinically significant weight gain (≥ 7%) 49% of patients experienced ↑ appetite relative to baseline and 35% required more food to reach satiety Weight gain was associated with ↑ frequency and quantity of food intake, ↑ preoccupation with food, and ↓ vigorous physical activity
Khazaal 2006a * [62]	Cross-sectional (case-control) Eating and weight related cognitions in patients with SCZ vs. HCs	40 SGA-treated SCZ outpatients Age: 33.8 40 HCs (matched for BMI) Age: 35.5 Two subgroups (*n* = 20) for each group: Overweight = BMI > 28 Comparison = BMI < 28	SCZ: 47.5% F HC: 52.5% F	Previous AP exposure (mean duration = 8.3 years): OLA, CLZ, QUE, RIS	Revised version of the Mizes Anorectic cognitive questionnaire (MAC-R)	Patients had ↑ total MAC-R and weight regulation subscale scores relative to controls Patients with BMI < 28 had ↑ MAC-R scores on all sub-scales compared to weight-matched controls Females had ↑ MAC-R scores than men
Khazaal 2006b * [68]	Cross-sectional (case-control) Binge eating symptomatology in overweight and obese patients with SCZ vs. HCs	40 SGA-treated SCZ outpatients Age: 33.8 40 HCs (matched for BMI) Age: 35.5 Two subgroups (*n* = 20) for each group: Overweight = BMI > 28 Comparison = BMI < 28	SCZ: 47.5% F HC: 52.5% F	NR	Binge eating/ED symptomatology (DSM-IV)	↑ prevalence of binge-eating symptoms and full BED in SCZ patients with BMI > 28 compared to weight-matched controls
Garriga 2019 [69]	Longitudinal (18 weeks) Effect of CLZ on food craving and consumption in patients with SMI	34 SMI patients SCZ: *n* = 27 Schizoaffective: *n* = 5 BD: *n* = 2 Age: 36.8 (range: 18–65) BMI: 27.3 Dosing: CLZ was initiated with a dose of 12.5–25 mg in the first day of treatment, followed by weekly upward adjustments of 25–50 mg (i.e., standard titration) Two subgroups: Normal weight (NW) = BMI < 25, *n* = 13 Overweight/obese (OWO) = BMI > 25, *n* = 21	38% F	Previous AP exposure (mean duration = 8.5 years): SGA = 28 (82.4%) FGA = 3 (8.8%) None = 3 (8.8%)	Food Craving (FCI, Spanish version) Cuestionario de Frecuencia de Consumo de Alimentos (CFCA)	No significant longitudinal changes in food craving or consumption, however moderating effects of baseline BMI and gender were observed In the NW group, ↑ cravings for “complex carbohydrates/proteins” and “simple sugar/trans fat” were associated with male gender and recent onset psychosis NW males had ↑ craving for “fast-food fats” at both time points compared to NW females In the OWO group, ↑ “fast-food fats” craving at Week 18 was associated with recent onset of psychosis In both groups, craving scores (FCI) were positively correlated with frequency of consumption (CFCA)
Karagianis 2009 [70]	Randomized, double blind, double dummy study (16 weeks) Effect of OLA on BMI, efficacy scores, weight and subjective appetite in patients with SMI	149 OLA-treated outpatients (115 completers) SCZ: *n* = 82 BD: *n* = 41 Schizoaffective: *n* = 15 Schizophreniform: *n* = 9 Other related disorder: *n* = 2 Age: 39 (range: 18–65) BMI: 28.1 Two treatment groups: Orally disintegrating OLA (ODO): *n* = 84 (MD: 13.87 mg/d) Standard OLA tablets (SOT): *n* = 65 (MD: 13.23 mg/d)	46% F 52.3% Caucasian, 33.6% Hispanic, 10.1% Black, 2.0% Asian, 1.3% First-nation, 0.7% Other	Previous AP exposure: 5–20 mg/day SOT (duration 4–52 weeks)	Hunger/appetite scale (VAS)	Patients in both groups experienced ↑ BMI, Patients in both groups experienced a non-significant trend towards ↓ hunger/appetite
Ryu 2013 [50]	Longitudinal (12 weeks) Effect of SGA treatment on eating behaviour in patients with SCZ	45 SCZ patients treated with SGA monotherapy OLA: *n* = 13 RIS: *n* = 24 ARI: *n* = 8 Age: 32.1 (range: 18–50)	50% F	Treated with current AP for 4–12 weeks (AP-free for 4 weeks prior to starting medication)	Binge eating/ED symptomatology (DSM-IV) Binary appetite/craving scale Food Craving (FCQ) DR-EBQ	BMI ↑ over time DR-EBQ total score was positively associated with weight gain and FCQ scores (“pre-occupation with food”, “loss of control”)
Smith 2012 [71]	Randomized trial (5 months) Effect of OLA and RIS on appetite in patients with chronic SCZ	46 SCZ inpatients OLA: *n* = 13 (MD: 25.2 mg/d) RIS: *n* = 17 (MD: 6.1 mg/d) Age: 41.2 (OLA), 42.5 (RIS)	2% F	All patients had been treated with multiple antipsychotics in the past	Hunger/appetite scale (VAS from 0–100) Eating Behavior Assessment (EBA)	Neither medication had a significant effect on appetite, with a non-significant trend towards ↓ appetite Both medications resulted in weight gain which was not correlated with changes in appetite (VAS) or eating behaviour (EBA)
Sentissi 2009 [72]	Cross-sectional (medication type) Effect of SGAs on eating behaviours and motivation in patients with SCZ	153 SCZ in- and outpatients SGA: *n* = 93 FGA: *n* = 27 Untreated: *n* = 33 Age: 33.1 (range: <50) BMI: 25.6 Among the untreated patients, 23 were AP-naïve, and 10 were AP-free for >3 months (mean duration: 7 months; range: 3–29 months)	38.6% F	SGA monotherapy: CLZ = 20 (MD: 374 mg/d) OLA = 23 (MD: 12 mg/d) AMI = 14 (MD: 571.4 mg/d) RIS = 20 (MD: 3.7 mg/d) ARI = 16 (MD: 11.9 mg/d) FGA: mainly HAL (*n* = 16, 59% of sample) or phenothiazines; MD = 289 mg/d (CPZ equivalents) Mean treatment duration (months): 36.2 (range = 3–86) Illness duration: 9.6 years	TFEQ DEBQ	↑ BMI was associated with ↑ TFEQ disinhibition (significant) and susceptibility to hunger (nearing significance) SGA-treated patients displayed ↑ reactivity to external eating cues (DEBQ) compared to FGA-treated, but not untreated patients Patients with both high restraint and high disinhibition were more likely to be overweight (significant) and to be treated with SGAs (nearing significance)
Abbas 2013 [73]	Cross-sectional (case-control) Food craving in OLA- or FGA-treated patients with SCZ vs. HCs	40 SCZ in- and outpatients OLA: *n* = 20 FGA: *n* = 20 Age (both): 39.4 (range: 18–65) BMI: 29.5 (OLA), 27.3 (FGA) 20 HCs (un-matched) Age: 40.9 BMI: 25.8	OLA: 45% F FGA: 55% F HC: 55% F	OLA (*n* = 20) or FGA (*n* = 20) for at least one month Flupentixol = 6 Zuclopenthixol acetate = 5 CPZ = 3 HAL = 2 Fluphenazine decanoate = 1 Pipotiazine palmitate = 1 Stelazine = 1 Trifluperazine = 1 Mean treatment duration (months): 15.1 (OLA), 19.7 (FGA)	Food Craving (FCI)	No significant difference in food craving between patients and controls
Blouin 2008 [74]	Cross-sectional (case-control) Adiposity and post-meal challenge eating behaviours in SGA-treated patients with SCZ vs. HCs	18 SCZ outpatients Age: 30.5 (range: 18–65) BMI: 28.8 20 HCs (matched for age and physical activity) Age: 29.5 BMI: 25.0	All Males	Previous AP exposure: FGA or SGA (mean duration = 35.3 months) Current SGA treatment: at least 3 months (mean duration = 24.6 months) OLA = 9 QUE = 3 CLZ = 2 RIS = 2 ZIP = 2	TFEQ Hunger/Appetite Scale (150-mm VAS) Food preference test and spontaneous intake (food weighed) 12 h fast prior to standardized breakfast, followed by an ad libitum buffet-type meal ~3 h later	Compared to HCs, patients reported ↑ subjective hunger and ↓ satiation following a standardized meal Compared to HCs, patients displayed ↑ TFEQ scores in all three domains which remained significant after controlling for BMI Among patients, susceptibility to hunger was positively associated with emotional susceptibility to disinhibition No significant group differences in spontaneous intake or food preference during ad libitum conditions
Folley 2010 [75]	Cross-sectional (case-control) Relative food preferences and hedonic judgements in SGA-treated patients with SCZ vs. HCs	18 SCZ outpatients treated with SGAs (MD: 93.6 mg/d) Age: 40.5 (range: 21–58) 18 HCs (matched for education and intelligence scores) Age: 38.9 (range: 20–52)	SCZ: 33% F HC: 44% F	Illness duration: 16.4 years	Food preference and food ratings task (Extra scanner task, 5-point Likert scale); participants tested prior to eating lunch	No significant group differences in response time or food preference during preference task Both patients and controls were more likely to give positive vs. neutral or negative ratings to food stimuli In patients, ↓ positive ratings were associated with ↑ anhedonia
Knolle-Veentjer 2008 [76]	Cross-sectional (case-control) Role of eating behaviour in body weight regulation in patients with SCZ vs. HCs	29 SCZ patients Paranoid subtype: *n* = 27 Disorganized subtype: *n* = 2 Age: 34 (range: 21–56) BMI: 26.8 23 HCs (matched for age, sex, educational level) Age: 32 (range: 20–58) BMI: 23.9	SCZ: 34.5% F HC: 26.1% F	QUE = 9 (MD: 600 mg/d) RIS = 8 (MD: 5.3 mg/d) OLA = 6 (MD: 15.83 mg/d) AMI = 4 (MD: 700 mg/d) ARI = 1 (MD: 20 mg/d) Flupentixol = 1 (MD: 10 mg/d)	FEV (German version of the TFEQ) Author-developed board game to assess delay of gratification using food reward Behavioral assessment of the dysexecutive syndrome (BADS)	Patients had ↓ delay of gratification, ↓ executive functioning (BADS), and ↑ BMI compared to controls In patients, ↑ FEV scores (disinhibition, restraint) were associated with ↓ executive functioning and ↑ BMI No significant difference in subjective appetite between patients and controls
Schanze 2008 [77]	Cross-sectional Comparing eating behaviours in patients with SCZ and MDD vs. HCs	42 SCZ inpatients Age: 33.6 BMI: 27.28 83 MDD inpatients Age: 40.42 BMI: 26.01 46 HCs (un-matched) Age: 35.7 BMI: 23.51	SCZ: 40.5% F MDD: 47% F HC: 47.8% F	SCZ (last 4 weeks): QUE = 6 RIS = 5 ZIP = 4 OLA = 2 CLZ = 1 SSRI = 1 None = 23 MDD (last 4 weeks): QUE = 1 RIS = 1 SSRI = 15 Mirtazapine = 10 None = 56	TFEQ	No significant group differences (patients vs. HCs, MDD vs. SCZ) for all TFEQ domains No significant effect of medication class (antidepressant, antipsychotic, no medication) on TFEQ scores
Roerig 2005 [78]	Randomized, double blind, parallel (2 weeks) Effect of OLA and RIS vs. placebo on eating behaviours in HCs	48 HCs, 18–60 years OLA: *n* = 16 (MD: 8.75 mg/d) RIS: *n* = 16 (MD: 2.875 mg/d) PLA: *n* = 16 Age: 33.6 (OLA), 36.2 (RIS), 32.7 (PBO) BMI: 23.6 (OLA), 25.0 (RIS), 24.1 (PBO)	OLA: 87.5% F RIS: 75% F PBO: 68.75% F	N/A	Hunger/appetite scale (100-mm VAS) Feeding laboratory (standardized breakfast, liquid lunch, ad libitum dinner where food was weighed) Resting energy expenditure (REE)	Participants in both AP groups gained weight, however only the OLA group reached statistical significance In the OLA group, weight gain was associated with a non-significant ↑ in food intake (kcal/day) and appetite compared to RIS or PBO None of the groups (OLA, RIS, PBO) showed a significant change in REE
Teff 2015 [79]	Randomized trial (12 days; 9 days of SGA exposure) Effect of acute SGA exposure on hunger and food intake in HCs	30 HCs OLA: *n* = 10 RIS: *n* =10 PBO: *n* = 10 Age: 26.1 (OLA), 25.9 (ARI), 29.9 (PBO) BMI: 22.1 (OLA), 22.4 (ARI), 21.8 (PBO) reported in [80]	30% F	N/A	Hunger/appetite scale (9-point Likert) Food intake weighed (objective) Activity level (number of steps)	Neither medication had a significant effect on weight, subjective hunger/fullness or calorie intake compared to PBO No significant change in physical activity levels in any group

Note: All main findings reported in this table are statistically significant unless otherwise indicated. SCZ = Schizophrenia, BD = Bipolar Disorder, MDD = Major Depressive Disorder, SMI = Severe Mental Illness, HCs = Healthy Controls, FEP = First Episode Psychosis, AMI = amisulpride, OLA = olanzapine, HAL = Haloperidol, RIS = Risperidone, QUE = Quetiapine, ZIP = Ziprasidone, CPZ = Chlorpromazine, PBO = Placebo, FGA = First generation antipsychotics, SGA = Second generation antipsychotic, NR = Not reported, TFEQ = Three-Factor Eating Questionnaire, FCQ = Food Craving Questionnaire, FCI = Food Craving Inventory, VAS = visual analog scale, DR-EBQ = Drug-Related Eating Behaviour Questionnaire, FFQ = Food Frequency Questionnaire, EBA = Eating Behaviour Assessment, DEBQ = Dutch Eating Behaviour Questionnaire, QEWP = Questionnaire on Eating and Weight Patterns, MD = Mean Dose, * = studies from the same cohort (Khazaal 2006a, 2006b).

**Table 6:** Characteristics of studies with neuroimaging methodologies.

Study	Design/Aim	Sample (Size, Diagnosis), Mean Age (Years), Mean BMI	Sex (% F), Race/Ethnicity	Mean Illness Duration/Previous AP Exposure (*n*)	Assessments	Main Significant Results
Stip 2015 * [84]	Longitudinal (16 week) study, pre- post with OLA administration Examining the salience network in SCZ patients on OLA treatment	15 SCZ patients not previously exposed to OLA switching to OLA Same cohort as 2012 study (authors did not specify switchers vs. AP-naïve, fasted)	NR	NR	fMRI (BOLD) during neutral vs. dynamic appetitive stimuli Hunger/appetite scale (VAS from 0–5) TFEQ	↓ Activation of SN (including ACC, aFI, and amygdala) in response to dynamic appetitive stimuli ↓ ACC and aFI activity were associated with ↑ ghrelin levels and ↓ dietary restraint (TFEQ)
Lungu 2013 * [83]	Cross sectional (case-control) Neuronal correlates of appetite regulation in patients with SCZ vs. HC (3 h since last meal)	25 SCZ (20 completers) AP (OLA excluded): *n* = 21 AP-naïve: *n* = 3 Age: 34.5 BMI: 26.62 11 HCs (10 included) Age: 35.2 BMI: 25.07	48% F SCZ: 24% F HC: 20% F	RIS = 12 QUE = 6 HAL = 2 CLZ = 1 Perphenazine = 1 No medication = 3	fMRI (BOLD) during neutral vs. static appetitive stimuli Hunger/appetite scale (VAS from 0–5) TFEQ	Common neuronal networks activated in both groups (left insula, primary sensory motor areas, and inferior temporal and parietal cortices) ↑ Responses to appetitive cues in areas of action planning and homeostatic signals (red thalamic nucleus, left parahippocampus, and left middle frontal gyrus) in SCZ group PANSS positive symptom scores positively correlated with activity in left middle frontal gyrus (action planning) and TFEQ disinhibition scores TFEQ restraint scores correlated positively with thalamic activity and negatively with disease severity (PANSS score) TFEQ hunger scores correlated negatively with parahippocampal activity and positively with AP dose (CPZ equivalents)
Stip 2012 * [85]	Longitudinal intervention (16 weeks) vs. HCs Evaluating neural changes associated with appetite in SCZ patients pre, and post OLA treatment vs. HCs (3 h since last meal)	24 SCZ patients not previously exposed to OLA (15 completers) Switch (no washout): *n* = 19 AP-naïve: *n* = 3 Age: 30.04 10 HCs Age: 33.9 Same cohort as Lungu 2013	SCZ: 21% F; 91.66% Caucasians, 8.33% Caribbean HC: 20% F; 100% Caucasian	RIS = 12 QUE = 6 HAL = 2 CLZ = 1 Perphenazine = 1 No medication = 3	fMRI (BOLD) during neutral vs. static appetitive stimuli Hunger/appetite scale (VAS from 0–5) TFEQ	OLA treatment ↑ weight gain significantly OLA treatment ↑ BOLD signal in response to appetitive stimuli in supplementary motor area, right fusiform gyrus, insular cortex, amygdala and parahippocampus OLA ↑ Neural activity in premotor area, somatosensory cortices and bilaterally in the fusiform gyri of patients to the same levels as HCs Hyperactivation (vs. HC) in 4 regions (insular cortices, amygdala, and cerebellum) in response to appetitive stimuli OLA-induced ↑ brain activity in response to appetitive stimuli was negatively corelated with TFEQ (dietary restraint) scores
Grimm 2012 [82]	Cross sectional (case-control) Striatal activation during appetitive cues (fasting state)	23 fasted (6h) chronic SCZ in- and outpatients on stable AP medication (MD: 346 mg/d CPZ equivalents) Age: 30.3 23 fasted HCs (matched for age, gender, parental SES, handedness) Age: 28.9	74% F	No change in the medication dose >25% or a switch to a different medication was allowed in the last 4 weeks SGA: *n* = 22 RIS = 4 OLA = 3 CLZ = 4 AMI = 3 QUE = 3 ARI = 3 ZIP = 1 FGA: *n* = 1 Flupenthixol = 1 Illness duration: 4.1 years	fMRI (BOLD) during neutral vs. static appetitive stimuli Hunger/appetite scale (VAS)	↓ Activation in the dorsal striatal region in patients Reduced activity remained significant after controlling for AP dose (CPZ equivalents) and body weight Patients and controls were similar in appetite ratings evoked by presentation of neutral or appetite stimuli
Emsley 2015 [88]	Prospective (13 weeks of AP treatment vs. HC) Morphological changes in brain regions associated with food intake regulation, metabolic parameters (BMI, fasting glucose, lipids) (fasting not specified)	22 AP-naïve FEP in- and outpatients randomized to receive RIS or flupenthixol decanoate long-acting injections (*n* not specified) SCZ: *n* = 13 Schizophreniform: *n* = 9 Age: 24.6 (range: 16–45) BMI: 22.1 23 untreated HCs (matched for age, sex, ethnicity, educational status) Age: 27	24% F FEP: 14% F; 64% mixed descent, 36% Black HC: 35% F; 70% mixed descent, 30% Black	No previous AP exposure; mean duration of untreated psychosis: 41 weeks Mean endpoint dose: 31.66 mg 2-weekly (RIS), 13.07 mg 2-weekly (flupenthixol)	Structural MRI changes in prespecified brain regions associated with hedonic and homeostatic body weight regulation	↑ Baseline left vDC volumes in patients compared to HCs Medicated FEP patients had ↓ vDC size (homeostatic), but no difference in prefrontal cortex (hedonic) size This volume change was also associated with ↑ BMI, dyslipidemia and elevated glucose in patients ↓ Volumes were not significant following post-hoc testing and were accompanied by ↑ volumes in control group
Borgan 2019 [86]	Cross-sectional (case-control) Neural responsivity to food cues in unmedicated first episode psychosis (fasting state used)	29 fasted (>12 h), untreated FEP patients SCZ: *n* = 27 Schizoaffective: *n* = 2 Age: 26.1 (range: 18–65) BMI: 25.2 28 fasted HCs (matched for age) Age: 26.4 BMI: 24.7	17% F FEP: 14% F; 12 White, 9 Black African or Black Caribbean, 6 Asian, 2 Mixed HC: 21% F; 10 White, 3 Black African or Black Caribbean, 11 Asian, 4 Mixed	Patients were AP-naïve or free from all psychotropic medication for at least 6 weeks Prior use = 20 AP-naïve = 9 Duration of prior treatment: 4.74 months Illness duration: 21.5 months	fMRI (BOLD signal) during neutral vs. static food cue (low and high calorie) IPAQ Dietary Instrument for Nutrition Education	↑ BOLD response in HCs vs. patients in the right insula, right anterior, posterior, medial and inferior orbitofrontal gyrus to food cues Comparing ROIs: ↑ Response to food cues in HCs in nucleus accumbens, but not in insula or hypothalamus In HCs, BMI was inversely correlated with mean BOLD signal in nucleus accumbens in response to food cues No group differences in neural responses to food cues between patients and HCs ↑ Fat consumption in patients than HCs in neural response to food cues
Mathews 2012 [87]	Interventional (pre- post 1-week OLA administration) open-label prospective design Neural activity associated with anticipation and receipt of food rewards after 1 week of OLA (in fasting state)	19 fasted (overnight) HCs Age: 27.5 (range = 18–50) BMI: 25.78 Dosing: 5 mg of OLA on the first night and 10 mg on the subsequent 6 nights	47.4% F 73.7% White, 10.5% African American, 10.5% Hispanic, 5.3% Mixed	N/A	fMRI (BOLD) during appetitive visual stimuli, and in response to receipt of cued food or water control (chocolate milk, tomato juice) Consumption of “liquid breakfast” measured post scan Hunger/appetite scale (5-point Likert) TFEQ	OLA ↑ Weight (1.1kg) OLA ↑ activation in regions for anticipatory reward (inferior frontal cortex, striatum, ACC) in response to visual food cues; ↑ activation of reward receipt regions (caudate, putamen); and ↓ activation in regions of inhibitory control of feeding (lateral orbital frontal cortex) OLA was associated with ↑ consumption of breakfast, and ↑ in disinhibited eating behavior (TFEQ)

Note: All main findings reported in this table are statistically significant unless otherwise indicated. SCZ = Schizophrenia, BP = Bipolar Disorder. HC = Healthy controls, FEP: First episode psychosis, AMI = amisulpride, OLA = olanzapine, HAL = Haloperidol, RIS = Risperidone, QUE = Quetiapine, ZIP = Ziprasidone, CPZ = Chlorpromazine, FGA = First generation antipsychotics, SGA = Second generation antipsychotic, NR = Not reported, TFEQ = Three-Factor Eating Questionnaire, FCQ = Food Craving Questionnaire, FCI = Food Craving Inventory, VAS = visual analog scale, DR-EBQ = Drug-Related Eating Behaviour Questionnaire, FFQ = Food Frequency Questionnaire, EBA = Eating Behaviour Assessment, DEBQ = Dutch Eating Behaviour Questionnaire, QEWP = Questionnaire on Eating and Weight Patterns; BOLD= blood oxygen level dependent response; ACC= anterior cingulate cortex, aFI= Anterior Fronto-insular, vDC= ventral Diencephalon, MD = Mean Dose, * = studies from the same cohort (Lungu 2013, Stip 2012, Stip 2015).

## Supplementary Materials

The following are available online at www.mdpi.com/xxx/s1, Table S1: Search strategy for Ovid MEDLINE electronic database search.
