# Supplementary material for: Exploring Patterns of Disturbed Eating in Psychosis: A Scoping Review
Source: Nutrients. 2020 Dec 18;12(12):3883. doi: 10.3390/nu12123883 (PMC7768542; doi:10.3390/nu12123883)
Supplement: Supplementary file 1 [file nutrients-12-03883-s001.pdf]

Supplementary Table S1: Search strategy for Ovid MEDLINE electronic database search

Database(s): **Ovid MEDLINE: Epub Ahead of Print, In-Process & Other Non-Indexed Citations, Ovid MEDLINE® Daily and Ovid MEDLINE®** 1946-Present

Search Strategy:

#	Searches
1	exp "schizophrenia spectrum and other psychotic disorders"/
2	(psychotic disorder* or schizo* or psychosis or psychotic* or first episode or antipsychotic naive or untreated or unmedicated).tw,kf.
3	Antipsychotic Agents/ or (antipsychotic* or neuroleptic*).tw,kf.
4	Chlorpromazine.mp. or Chlorpromazine/
5	Chlorprothixene.mp. or Chlorprothixene/
6	Droperidol.mp. or Droperidol/
7	Flupentixol.mp. or Flupenthixol/
8	Fluphenazine.mp. or Fluphenazine/
9	Haloperidol.mp. or Haloperidol/
10	(Levomepromazine or Methotrimeprazine).mp. or Methotrimeprazine/
11	Loxapine.mp. or Loxapine/
12	Mesoridazine.mp. or Mesoridazine/
13	Molindone.mp. or Molindone/
14	Periciazine.mp.
15	Pimozide.mp. or Pimozide/
16	Prochlorperazine.mp. or Prochlorperazine/
17	Promazine.mp. or Promazine/
18	Thioproperazine.mp.
19	Thioridazine.mp. or Thioridazine/
20	Thiothixene.mp. or Thiothixene/
21	Trifluoperazine.mp. or Trifluoperazine/
22	(Zuclopenthixol or Clopenthixol).mp. or Clopenthixol/
23	(antipsychotic* adj5 (typical or first generation)).tw,kf.
24	(amisulpride or solian).mp.
25	ARIPIPRAZOLE/ or (aripiprazol or aripiprazole or abilify or OPC 14597).mp.

26	(asenapine or saphris or sycrest).mp.
27	(blonanserin or lonasen).mp.
28	Brexpiprazole.mp.
29	CLOZAPINE/ or (cloazpine or clozaril or leponex).mp.
30	(iloperidone or fanapt or fanapta).mp.
31	Lurasidone Hydrochloride/ or (lurasidone or latuda or sm 13496).mp.
32	(melperone or buronil).mp.
33	(olanzapine or zyprexa).mp.
34	Paliperidone Palmitate/ or (paliperidone or invega or r 76477).mp.
35	(perospirone or lullan).mp.
36	quetiapine fumarate/ or (quetiapine or seroquel).mp.
37	remoxipride/ or (remoxipride or roxiam).mp.
38	risperidone/ or (risperidone or risperdal).mp.
39	(sertindole or serdolect or serlect).mp.
40	sulpiride/ or (sulpiride or dogmatil).mp.
41	(ziprasidone or geodon).mp.
42	(antipsychotic* adj5 (atypical or second generation)).tw,kf.
43	1 or 2 or 3 or 4 or 5 or 6 or 7 or 8 or 9 or 10 or 11 or 12 or 13 or 14 or 15 or 16 or 17 or 18 or 19 or 20 or 21 or 22 or 23 or 24 or 25 or 26 or 27 or 28 or 29 or 30 or 31 or 32 or 33 or 34 or 35 or 36 or 37 or 38 or 39 or 40 or 41 or 42
44	exp Neurobiology/
45	exp Magnetic Resonance Imaging/
46	(functional magnetic resonance imag* or fMRI or functional MRI).tw,kf.
47	exp Functional Neuroimaging/
48	(functional imag* or neuroimag*).tw,kf.
49	((neurochemical or h?emodynamic or blood oxygen or BOLD) adj3 (change* or response*)).tw,kf.
50	exp Food Preferences/ or food preference*.tw,kf.
51	(food cue* or appetite provoking cue*).tw,kf.
52	(neural correlate* or neural response*).tw,kf.
53	44 or 45 or 46 or 47 or 48 or 49 or 50 or 51 or 52
54	"surveys and questionnaires"/ or self report/
55	Awareness/ or Weight Perception/

56	(survey or self-report or questionnaire).tw,kf.
57	(Leeds Food Preference Questionnaire or LFPQ or Dietary Instrument for Nutrition Eduction or Three Factor Eating Questionnaire or TFEQ or Yale Food Addiction Scale or YFAS or Semi-quantitative Food Frequency Questionnaire or FFQ).mp.
58	54 or 55 or 56 or 57
59	Ventral Tegmental Area/ or (ventral tegment* or VTA).tw,kf.
60	exp Basal Ganglia/
61	(basal ganglia or BG or nucleus accumbens or NAc or ventral striat*).tw,kf.
62	Motivation/ or Drive/ or Craving/
63	(motivation* or incentiv* or drive or craving).tw,kf.
64	Anhedonia/ or (anhedoni* or hedoni*).tw,kf.
65	((Mesolimbic or reward or hedonic) adj3 (system or pathway)).tw,kf.
66	((relative or food or anticipation) adj3 reward).tw,kf.
67	exp Hypothalamus, Middle/ or (arcuate nucleus or ARC or ventromedial nucleus or VMN).tw,kf.
68	((dopamin* or seroton*) adj3 (system or circuit* or pathway)).tw,kf.
69	Orexins/ or Orexin Receptors/ or (orexin* or orexigen*).tw,kf.
70	59 or 60 or 61 or 62 or 63 or 64 or 65 or 66 or 67 or 68 or 69
71	appetite/ or appetite.tw,kf.
72	dietary profile*.tw,kf.
73	eating/ or eating.tw,kf.
74	energy balance.tw,kf.
75	energy intake/ or energy intake.tw,kf.
76	Food Preferences/ or food preference*.tw,kf.
77	feeding behavio?r*.tw,kf.
78	"feeding and eating disorders"/ or ((feeding or eating) adj2 disorder*).tw,kf.
79	food addiction/ or food addiction*.tw,kf.
80	(food consum* or (food adj2 seeking)).tw,kf.
81	Hunger/ or (hunger or hungry).tw,kf.
82	hyperphagia/ or hyperphag*.tw,kf.
83	nutritional status/ or nutritional status.tw,kf.
84	(nutrient adj2 (composition* or profile*)).tw,kf.
85	obesity/ or obesity.tw,kf.

86	bulimia/ or bulimia nervosa/ or (bulimia or BN).tw,kf.
87	Binge-Eating Disorder/ or (BED or binge eat* or compulsive eat*).tw,kf.
88	energy intake/ or portion size/ or serving size/
89	71 or 72 or 73 or 74 or 75 or 76 or 77 or 78 or 79 or 80 or 81 or 82 or 83 or 84 or 85 or 86 or 87 or 88
90	43 and (53 or 70) and 89
91	90
92	limit 91 to humans
